# Pregnancy intentions by sexual orientation among pregnancies across the life course

**DOI:** 10.1093/aje/kwag165

**Published:** 2026-11-04

**Authors:** Payal Chakraborty, Corinne H. Rocca, Colleen A. Reynolds, Isa Berzansky, Kodiak R. S. Soled, Sarah McKetta, Ange-Marie Hancock, Danielle Bessett, Sebastien Haneuse, Brittany M. Charlton

**Affiliations:** 1 Department of Social Medicine, School of Medicine, University of North Carolina at Chapel Hill, Chapel Hill, NC, United States; 2 Advancing New Standards in Reproductive Health, Department of Obstetrics, Gynecology and Reproductive Sciences, School of Medicine, University of California San Francisco, Oakland, CA, United States; 3 Harvard Pilgrim Health Care Institute, Boston, MA, United States; 4 Department of Epidemiology, Harvard T.H. Chan School of Public Health, Boston, MA, United States; 5 Department of Population Medicine, Harvard Medical School, Boston, MA, United States; 6 Department of Epidemiology, Columbia University, New York, NY, United States; 7 Department of Political Science, College of Arts and Sciences, The Ohio State University, Columbus, OH, United States; 8 Department of Sociology, University of Cincinnati, Cincinnati, OH, United States; 9 Department of Biostatistics, Harvard T.H. Chan School of Public Health, Boston, MA, United States

**Keywords:** pregnancy intentions, sexual orientation, induced abortions, United States, Canada

## Abstract

Sexual minority (SM) people may be at increased risk of “unintended” pregnancies, but limited research has examined potential disparities. We examined the intention status of lifetime pregnancies by sexual orientation. We pooled data from the Nurses’ Health Study 3 and Growing Up Today Study (*N* = 36 967 pregnancies 1978-2024). Using multinomial models, we estimated relative risk ratios (RRRs) for pregnancy intention (actively trying/wanted then or sooner, not trying but glad, wanted later but not then, and unwanted then and in the future) among SM groups (completely heterosexual with same-sex experience, mostly heterosexual, bisexual, and lesbian/gay) vs completely heterosexual participants with no same-sex experience. Model-predicted probabilities show that 15%-33% of pregnancies within each sexual orientation group were wanted later/unwanted. Compared to pregnancies to completely heterosexual participants with no same-sex experience, all SM groups had more pregnancies that were wanted later (RRRs from 1.22 [completely heterosexual with same-sex experience] to 2.28 [bisexual]) and unwanted then and in the future (RRRs ranging from 1.74 [completely heterosexual with same-sex experience] to 5.23 [bisexual participants]), compared to pregnancies occurring when participants were actively trying/wanted the pregnancy then or sooner. Our findings suggest that pregnancies among SM groups disproportionately are unwanted or occur too early.

## Introduction

The ability of individuals to freely make decisions about if, when, and how to form families is a fundamental aspect of health and well-being. However, misalignment between individuals’ pregnancy desires and pregnancy outcomes is common in the United States. For example, as of 2019, an estimated 42% of pregnancies in the United States are considered “unintended” (defined as having occurred earlier than desired or when not desired at all).^1–3^ Furthermore, access to the resources and support to exercise reproductive autonomy is not equitably distributed; thus, systemically marginalized groups, including those with fewer financial resources and educational opportunities, disproportionately experience unintended pregnancies.^1,2^ Sexual minority (SM) individuals (eg, those who identify as lesbian, gay, or bisexual, or have same-sex/gender attractions or partners) are known to experience several barriers—including but not limited to socioeconomic barriers, lack of access to desired contraception, gaps in sexual education, experiences of stigma and discrimination in the community and in health care settings, and experiences of interpersonal violence—which can lead to pregnancy when it is not desired.^4–7^ Emerging literature has begun to examine disparities related to unintended pregnancy by sexual orientation,^8–10^ but this area of research is understudied.

Studying pregnancy intentions among SM populations poses unique challenges. First, the few datasets that collect information on sexual orientation are cross-sectional,^8–10^ treating sexual orientation as a static variable.^11–13^ However, sexual orientation is multidimensional (consisting of identity, attractions, and behavior), and almost no dataset includes measures of all 3 of these dimensions.^14^ Lack of these detailed measures precludes our understanding of the disparate effects of minority stress, which operates differentially among different SM groups, leading to heterogeneous health outcomes.^9,15–19^ Second, pregnancy intentions are complex and multifaceted.^20–22^ Yet, most analyses treat pregnancies as having been either “intended” vs “unintended”, categories that overly simplify the range of individuals’ feelings about pregnancy. Finally, most data sources with information about pregnancy intentions do not include information about sexual orientation.

We used data from the Nurses’ Health Study 3 (NHS3) and the Growing Up Today Study (GUTS), 2 cohort studies including multidimensional measures of sexual orientation, to quantify sexual orientation differences in the proportion of pregnancies in which the participants reported they: (1) were actively trying to become pregnant or wanted to be pregnant then or sooner, (2) were not actively trying but glad to become pregnant, (3) wanted to be pregnant at some point in the future but not then, and (4) did not want to be pregnant then or anytime in the future. Furthermore, because induced abortion is a central health care mechanism through which individuals can align pregnancy outcomes with their intentions, examining abortion utilization provides critical insight into how effectively different sexual orientation groups are able to exercise reproductive autonomy. Moreover, structural barriers to abortion care are experienced differently across sexual orientation groups.^23^ Therefore, we also examined whether induced abortion utilization by sexual orientation differed across strata of intention.

## Methods

### Data source

NHS3 is an ongoing cohort (with open enrollment) of nurses and nursing students residing in the United States or Canada. Recruitment began in 2010 and includes individuals born on or after January 1, 1965. Participants complete online questionnaires approximately every 6 months. GUTS consists of the children of Nurses’ Health Study II participants, with enrollment completed in 2 waves (1996 and 2004). Since baseline, participants have been followed with questionnaires every 2 years, and beginning in 2013, all participants have been followed together.

### Lifetime pregnancies and pregnancy intention

In both cohorts, participants reported their lifetime pregnancies and, retrospectively, their intentions for each pregnancy. In NHS3, pregnancies before baseline were reported on the first questionnaire and pregnancies after baseline on the 13th questionnaire. Minor changes were made to the baseline questionnaire over time, resulting in different versions (eg, first questionnaire version 1). Pregnancy intention was assessed on the first questionnaire versions 4-5 and on the 13th questionnaire. Participants who completed the earlier first questionnaire (ie, versions 1-3) were invited to a catch-up survey; intention was assessed for pregnancies if they completed the catch-up survey and either confirmed or corrected their prior pregnancy grid, as well as for any new pregnancies reported there (Figure S1). In GUTS, lifetime pregnancy history and intentions for each pregnancy were assessed in 2019 (Figure S1).

The intention question asked participants to think back about how they felt about each pregnancy. We analyzed 4 intention categories: (1) actively trying or wanting to be pregnant then or sooner (shorthand: actively trying/wanted then or sooner), (2) not actively trying but being glad to become pregnant (shorthand: not trying but glad), (3) wanting to be pregnant at some point in the future but not then (shorthand: wanted later but not then), and (4) not wanting to be pregnant then and anytime in the future (shorthand: unwanted then and in the future). The questions included in each survey are listed in Table S1. In this manuscript, we use “pregnancy intention” as an umbrella term for these 4 categories, while recognizing that they combine multiple constructs. Specifically, the response options combine several dimensions of pregnancy preferences: (1) pregnancy wantedness at any time, (2) desired timing of the pregnancy, and (3) emotional orientation upon pregnancy recognition. For example, the “not trying but glad” category primarily reflects a positive emotional response to a pregnancy that was not actively planned, whereas “wanted later but not then” captures the presence of pregnancy desire coupled with mistimed occurrence. We therefore treat the 4 categories as distinct, instead of collapsing them into intended, mistimed, and unwanted categories. In addition, because these reports are retrospective, they may also reflect *post hoc* meaning-making and adaptation to the pregnancy experience, which we consider part of the construct captured by this measure. Furthermore, because pregnancy intentions were retrospectively reported, and because reporting of pregnancy intentions may change over time,^24^ we examined the distribution of pregnancy intentions by time between the pregnancy and reporting of intentions.

### Pregnancy outcomes

For each pregnancy, participants reported the pregnancy outcome (eg, live birth, stillbirth, miscarriage, or induced abortion). Using these data, we examined whether pregnancies ended in an induced abortion compared to any other pregnancy outcome.

### Sexual orientation

Sexual orientation identity was assessed in both NHS3 and GUTS using an item adapted from the Minnesota Adolescent Health Survey, which asked, “which of the following best describes your feelings?” with the following response options: “completely heterosexual (attracted to persons of the opposite sex),” “mostly heterosexual,” “bisexual (equally attracted to men and women),” “mostly homosexual,” and “completely homosexual (gay/lesbian, attracted to persons of the same sex).”^25^ NHS3 also included additional questions about attractions, and both NHS3 and GUTS included questions about sexual partnerships. This measure was repeatedly asked in the 5th, 10th, and 13th NHS3 questionnaires, and in nearly all the GUTS questionnaires. We combined data on identity, partners, and attractions to construct 5 sexual orientation groups:

1. Completely heterosexual with no same-sex experience (reference).
2. Completely heterosexual with same-sex experience.
3. Mostly heterosexual.
4. Bisexual.
5. Lesbian/gay.

In NHS3, group 1 consisted of those who identified as completely heterosexual and reported no same-sex/gender or nonbinary partners/attractions, and never previously identified with a SM identity. In GUTS, this group consisted of those who identified as completely heterosexual and reported no same-sex partners. In NHS3, group 2 consisted of those who identified as completely heterosexual and reported same-sex/gender or nonbinary partners/attractions, or previously identified with a SM identity. In GUTS, this group consisted of those who identified as completely heterosexual and reported same-sex partners. In both NHS3 and GUTS, group 5 consisted of those who identified as mostly homosexual, completely homosexual, or lesbian/gay. Additional details about the coding of sexual orientation in both of these cohorts have been published elsewhere.^26,27^

For NHS3, we used the measure of sexual orientation that was assessed closest in time to the pregnancy; the measure could have been administered prior to or after the pregnancy. For GUTS, because sexual orientation was asked more often, we used the closest measure prior to the pregnancy.

### Covariates

We included 3 baseline covariates that may affect participants’ sexual orientation identity development or disclosure and pregnancy outcomes: participants’ year of birth, race/ethnicity, and the US Census region of residence at birth (plus non-US).

### Statistical analysis

Analyses pooled NHS3 and GUTS data and were conducted at the pregnancy level. The eligible sample included participants who reported at least 1 pregnancy and had non-missing data on sexual orientation and intention status of the pregnancy. While there were missing data on intention because the variable was not assessed for all pregnancies, very few observations (<0.5%) were missing data on intention due to nonresponse (Table S2). To account for missing data for covariates, we performed multiple imputation using multivariate imputation by chained equations.

We used multinomial logistic regression models to estimate relative risk ratios (RRRs) (and 95% CIs) of intention status of pregnancies by sexual orientation. We fit these models using generalized estimating equations (GEEs) to account for multiple pregnancies per person. We used weights that were the product of stabilized inverse probability weights (IPWs) to account for confounding by covariates multiplied by weights equal to inverse cluster sizes to account for informative clustering, as the intention status of pregnancies could be related to the number of lifetime pregnancies.^28^ We generated model-based predicted probabilities of each intention status by sexual orientation groups. We fit these multinomial weighted GEE models using SAS 9.4. Then, across the 4 strata of pregnancy intentions, we examined differences in induced abortion by sexual orientation. We used separate log-linear models fit via GEEs with IPWs and inverse cluster size weights to estimate risk ratios (RRs). We additionally presented sensitivity analyses with crude estimates and estimates adjusted for time (years) between pregnancy occurrence and reporting. We used R version 4.2.0 to fit these models.

## Results

### Sample characteristics

The analytic sample included 36 967 pregnancies among 15 111 participants (Table 1). Over 90% of pregnancies were to White non-Hispanic participants. Participants who were born in the US Midwest and Northeast regions contributed the most pregnancies, and about 5% of pregnancies were to those who were born outside of the United States.

Overall, 78% of pregnancies were among completely heterosexual participants with no same-sex experience, followed by 13% among mostly heterosexual, 5% among completely heterosexual with same-sex experience, 2% among bisexual, and <1% among lesbian/gay participants. Among all pregnancies, 66% were pregnancies in which participants were actively trying or wanted the pregnancy then or sooner, 19% in which participants were not trying but glad to become pregnant, 12% in which participants wanted the pregnancy later but not then, and 4% in which participants did not want the pregnancy then or in the future. Distributions of sexual orientation and pregnancy intentions were similar in both cohorts (Table S3).

### Pregnancy intentions by sexual orientation

Pregnancies to completely heterosexual participants with no same-sex experience had model-predicted probabilities of 17%, 11%, and 4% of being in the “not trying but glad,” “wanted later but not then,” and “unwanted then and in the future” categories, respectively (Table 2).

Compared to pregnancies to completely heterosexual participants with no same-sex experience, pregnancies to completely heterosexual participants with same-sex experience were more likely to be “wanted later but not then” (12% vs 11%; RRR, 1.22; 95% CI, 0.95-1.56) and “unwanted then and in the future” (6% vs 4%; RRR, 1.74; 95% CI, 1.18-2.55) vs in the “actively trying/wanted then or sooner” category (64% vs 68%).

Mostly heterosexual participants showed a similar trend as completely heterosexual participants with same-sex experience but with slightly higher magnitudes. Compared to pregnancies to completely heterosexual participants with no same-sex experience, pregnancies to mostly heterosexual participants were more likely to be “wanted later but not then” (16% vs 11%; RRR, 1.67; 95% CI, 1.44-1.95) and “unwanted then and in the future” (7% vs 4%; RRR, 2.40; 95% CI, 2.92-3.01) vs in the “actively trying/wanted then or sooner” category (61% vs 68%).

Differences in the intention status of pregnancies were magnified for bisexual participants. Compared to pregnancies to completely heterosexual participants with no same-sex experience, pregnancies to bisexual participants were more likely to be “wanted later but not then” (19% vs 11%; RRR, 2.28; 95% CI, 1.65-3.15) and “unwanted then and in the future” (14% vs 4%; RRR, 5.23; 95% CI, 3.52-7.78) vs in the “actively trying/wanted then or sooner” category (52% vs 68%).

Lesbian participants showed a different trend. Compared to pregnancies to completely heterosexual participants with no same-sex experience, pregnancies to lesbian/gay participants were less likely to be in the “not trying but glad” category (7% vs 17%; RRR, 0.40; 95% CI, 0.19-0.81). Furthermore, pregnancies to lesbian/gay participants were almost 3 times as likely to be in the “unwanted then and in the future” category (10% vs 4%; RRR, 2.94; 95% CI, 1.61-5.36).

Estimates from the crude model were similar to those in the main model (Table S4). In analyses of the distribution of pregnancy intentions by the time between pregnancy occurrence and reporting, as the years between pregnancy and reporting increased, fewer pregnancies were reported in the “actively trying/wanted then or sooner” category, and more pregnancies were reported in all other categories (Table S5). This trend was similar in all sexual orientation groups. Estimates adjusting for time between pregnancy occurrence and reporting were similar to the main findings (Tables S6 and S7).

### Induced abortions by strata of pregnancy intentions among pregnancies by sexual orientation

Overall, 7% of pregnancies ended in induced abortion (Table 1). Among the “actively trying/wanted then or sooner” and the “not trying but glad” categories, very few pregnancies ended in induced abortion in all sexual orientation groups (Table 3). Among pregnancies that were wanted later but not then, model-predicted probabilities showed that 44% to 49% ended in induced abortions. Those in the SM groups were more likely to end in induced abortions compared to those to completely heterosexual participants with no same-sex experience (RRs ranged from 1.22 to 1.38). Among pregnancies that were unwanted then and in the future, 46% to 65% ended in induced abortions. Those to mostly heterosexual (RR, 1.22; 95% CI, 1.08-1.39) and bisexual (RR, 1.28; 95% CI, 1.03-1.58) participants were more likely to end in induced abortions compared to completely heterosexual participants with no same-sex experience, and those to completely heterosexual participants with same-sex experience and lesbian/gay participants were just as likely to end in induced abortions.

## Discussion

Using data from 2 national cohorts, we examined differences in intentions among pregnancies by sexual orientation and induced abortions within strata of intentions. We found that a range of 15%-33% of pregnancies within each sexual orientation group were wanted later but not then or unwanted then and in the future. However, pregnancies among SM groups, and particularly among the bisexual group, were disproportionately unwanted or too early. Pregnancies among the lesbian group were disproportionately unwanted but not too early. This work is novel given that no studies have been able to disaggregate pregnancies that were planned and wanted, were not planned and wanted, occurred too soon, and were unwanted then and in the future by sexual orientation. Understanding misalignment between pregnancy experiences and pregnancy desires by sexual orientation is increasingly vital, as reproductive health care access declines and discriminatory legislation affecting SM populations intensifies across the United States,^29,30^ which may further widen SM disparities.

Our findings are consistent with previous studies which found that some SM groups have a higher proportion of pregnancies that are “unintended” compared to their heterosexual peers.^8,10^ Only one other study examined these differences in pregnancy intentions at the pregnancy level, finding that, compared to pregnancies to heterosexual women who have sex with men, pregnancies to heterosexual women who have sex with women, bisexual women, and lesbian women have a higher risk of being both mistimed and unwanted.^8^ Although the way pregnancy intentions were measured in our study and previous studies were different, our findings were similar, as we found that pregnancies among all SM groups were more likely to be wanted later but not then and unwanted then and in the future. We additionally describe pregnancy intentions among an additional group, mostly heterosexual individuals, who also have higher proportions of pregnancies that were wanted later but not then and unwanted then or in the future, and lower proportions of pregnancies where participants reported actively trying or that they wanted the pregnancy then or sooner, which has never been described before.

Current studies of pregnancy intentions by sexual orientation (including this one) do not actually measure participants’ true risk of having an “unintended” pregnancy.^31,32^ Instead, they ask about participants’ pregnancies that already occurred and assess whether the pregnancy was intended or unintended. However, this question is different from estimating the true risk of unintended pregnancy that assesses: “out of all the people who did not want to be pregnant, how many actually became pregnant?” Because of this limitation, we do not know whether the documented differences between sexual orientation groups are due to different numbers of people in each group who do not want to be pregnant, or different probabilities of becoming pregnant among people who do not want to be pregnant. Therefore, the current available data do not allow the examination of whether SM people are more likely to experience an “unintended” pregnancy than heterosexual people. To measure the true risk, we would need a prospective cohort study where we know the pregnancy preferences or intentions of all participants, including those who do not go on to experience pregnancy.^31,32^

Nevertheless, several factors may contribute to a higher proportion of “unintended” pregnancies among SM individuals, many of which stem from structural, interpersonal, and individual stigma. For example, some SM women have sex with men including to conform to heterosexual norms and avoid stigma.^33,34^ This occurs particularly in adolescence,^35^ and may contribute to higher risks in unwanted pregnancies. SM individuals have an earlier sexual debut than their heterosexual peers,^36^ which can lead to an “unintended” pregnancy.^37,38^ SM individuals have higher teen pregnancy rates than heterosexual peers,^5,35^ many of which are “unintended”. SM individuals are also more likely than heterosexual individuals to experience sexual assault,^8,39–43^ which can lead to an “unintended” pregnancy.^35,44–47^ Furthermore, SM individuals have poorer access to healthcare, particularly reproductive healthcare, which may limit access to contraception.^6^ Among those who receive care, SM individuals are less likely to receive contraceptive counseling because many providers erroneously assume that SM individuals engage in sexual activity that does not put them at risk for pregnancy.^4,7^ While these pathways to “unintended” pregnancies are well documented among bisexual and lesbian individuals, little is known about the pathways leading to unintended pregnancies among heterosexual individuals with same-sex experience and mostly heterosexual individuals. These are large SM groups who experience unique forms of stigma and adverse health outcomes^15,16,48^ and who are commonly misclassified into the reference group.

Moreover, SM stress operates in both shared and distinct ways across SM subgroups—not just by identity but also across partnerships—contributing to heterogeneity in unintended pregnancy experiences.^49,50^ For example, individuals who identify as completely heterosexual but report same-sex experiences may experience dissonance between their sexual identity and behavior. “Mostly heterosexual” is a distinct but often unrecognized SM identity^15,48^; these individuals may experience identity invalidation and some minority stressors stemming from monosexist discrimination that are more similar to those experienced by bisexual individuals, such as identity erasure and less social support.^15^ Completely and mostly heterosexual people with same-sex experience may also share unique internal stressors and discrimination when in a queer-presenting relationship, compared with other SM groups. Bisexual individuals often face more discrimination than other SM groups, including experiencing stigma from both heterosexual and gay/lesbian communities.^51^ Lesbian and gay individuals in same-sex/gender partnerships may be more open about their sexual identity but face more overt discrimination and exclusion from reproductive health services that are framed around heterosexual norms and assumptions.^7^

These minority stressors can lead to internalized stigma, adverse mental health, relationship instability, barriers to consistent contraceptive use, and other factors that can affect pregnancy risk.^18,19^ Notably, these experiences may also have changed across time and sociopolitical climate, as shifts in legal protections, public attitudes, and community visibility can both mitigate some forms of stigma (eg, new policy protections) and intensify others (eg, backlash or policy rollbacks).^29^ Over time, growing social acceptance has also enabled more individuals to disclose an SM identity, which may alter both their minority stress and pregnancy experiences.^52,53^ Overall, unique experiences of distal (eg, discrimination) and proximal (eg, concealment, internalized stigma) minority stressors across SM groups may shape differential exposure to pregnancy risk and influence whether and how unintended pregnancies occur and are managed.^18,19^

We found higher proportions of pregnancies ending in abortion among some SM groups across all intention strata. For example, among pregnancies where participants were actively trying or wanted to be pregnant at that time or sooner—while all sexual orientation groups had low abortion use—pregnancies to some SM groups had a higher proportion of induced abortions than completely heterosexual people with no same-sex experience. This finding may be explained by studies that have documented that SM people have higher risks of pregnancy complications,^54^ and so they may be more likely to need life-saving abortion care. We also found that pregnancies to SM people are more likely to end in induced abortions among pregnancies that were wanted later but not then and unwanted then and in the future. This finding of higher use of abortion among SM populations in this study may be specific to this sample, as the participants are nurses and nursing students or children of nurses, who have greater financial resources, health literacy, health care access, and ability to navigate the health care system than the general population. Despite our findings, prior studies have shown that SM people encounter greater barriers to accessing abortion care than their heterosexual peers, including financial constraints, transportation challenges due to having to travel far and attend multiple visits, psychosocial challenges, and experiences of discrimination in reproductive health care settings.^7,23^

“Unintended” pregnancy rates have been historically used as indicators of reproductive autonomy and reproductive health care access.^55,56^ However, this approach has 2 main pitfalls. First, pregnancy intentions are hard to measure. Most available data use a simple “intended” vs “unintended” classification, which fails to reflect the complex and multidimensional nature of people’s feelings about pregnancy.^20–22^ Intentions can be ambivalent; influenced by shifting personal, social, and structural factors like economic hardship or intimate partner violence; and may evolve over the course of the pregnancy.^20,21^ Second, public health and clinical strategies to reduce “unintended” pregnancies have not always led to greater reproductive autonomy; instead, “unintended” pregnancy is frequently portrayed as the result of relying on less effective (or no) contraceptive methods, an approach that can contribute to stigmatization and blame.^56^ Thus, other more patient-centered approaches have emerged, such as measures capturing pregnancy and childbearing preferencess^22^ and measures that capture the acceptability of pregnancy after someone has found out they are pregnant.^21^ Future studies, including existing cohorts, such as those used in this study, would benefit from adopting these recently developed measures to better align research questions related to pregnancy preferences with implications for reproductive autonomy.

Among the study limitations, pregnancy intention was measured retrospectively, which may lead to underreporting of unintended pregnancies, as individuals may reassess or reinterpret their pregnancy intentions over time, particularly if the pregnancy resulted in a live birth.^24^ In our analysis of intention distributions by the time interval between pregnancy and reporting, we observed that as this interval increased, the proportion of pregnancies in the “actively trying” category decreased, and the proportions in the “not trying but glad,” “wanted later but not then,” and “unwanted then and in the future” increased (Table S7). This pattern is opposite our expectations, given prior evidence that individuals tend to retrospectively rationalize their pregnancies as “intended” even when they were originally “unintended” over time.^24^ Pregnancies reported after longer intervals occurred further in the past, making it difficult to disentangle the extent to which these differences reflect misreporting vs underlying differences related to the time period in which the pregnancies occurred. Notably, this pattern was non-differential by sexual orientation. Additionally, the response categories combine several dimensions of pregnancy preferences, and while the middle intention categories capture timing, they are not mutually exclusive categories. Second, this study may have limited generalizability due to the higher socioeconomic status and lower racial/ethnic diversity in the cohorts compared to the general population. Because the NHS3 cohort consists of nurses and nursing students, and the GUTS cohort consists of children of nurses, these cohorts are more privileged and have higher health literacy than the general population. Thus, the burden estimated in this study is likely to be a conservative estimate of the burden in the general population. Indeed, our estimates of unwanted pregnancies were lower than those from the general population.^57^ Third, because abortions are self-reported in this study, they are likely underreported.^58^ There are 2 ways that abortions could be underreported in the data: (1) by participants not reporting the pregnancy at all, and (2) by reporting the outcome of the pregnancy differently, for example, as a miscarriage. It is unclear to what extent they are underreported because of the lack of national estimates comparable to the data structure in our study (ie, the proportion of pregnancies across people’s lifetimes that ended in induced abortions) and populations with greater SES are also known to have lower abortion rates due to greater financial resources and access to health care to prevent pregnancy when it is not desired.^59,60^ Fourth, the underreporting of abortions may be differential by sexual orientation, though the direction of potential differential underreporting is unknown. For example, someone who discloses a stigmatized identity may be more likely to report utilizing health care that is stigmatized. They may also be less likely to report using stigmatized health care to avoid further stigma. Despite the above limitations, there is limited availability of large datasets that include comprehensive measures of sexual orientation and pregnancy intentions as well as longitudinal measurements of these variables.

Overall, we found that pregnancies among all SM groups were more likely to be wanted sometime in the future but not then or unwanted then and in the future, compared to pregnancies among completely heterosexual individuals with no same-sex experience. These risks varied by group, with bisexual individuals experiencing a particularly elevated burden. Our findings underscore the need to understand misalignment between individuals’ pregnancy desires and the pathways that lead to differential misalignment by sexual orientation, as well as to ensure that everyone—regardless of sexual orientation—is able to attain the childbearing outcomes they desire.

## Supplementary Material

Supplement

Supplementary material is available at the *American Journal of Epidemiology* online.

## Figures and Tables

**Table 1:** Characteristics of participants experiencing each pregnancy reported in the Nurses’ Health Study 3 (NHS3) and Growing Up Today Study (GUTS).^a^

	Completely heterosexual with no same-sex experience	Completely heterosexual with same-sex experience^b^	Mostly heterosexual	Bisexual	Lesbian/gay	Overall

	(*n* = 28 981, % = 78.4)	(*n* = 1931, % = 5.2)	(*n* = 4961, % = 13.4)	(*n* = 795, % = 2.2)	(*n* = 299, % = 0.8)	(*n* = 36 967, % = 100.0)
Birth year of pregnant person, *n* (%)						
1965-1969	4857 (16.8%)	143 (7.4%)	507 (10.2%)	64 (8.1%)	57 (19.1%)	5628 (15.2%)
1970-1974	4842 (16.7%)	186 (9.6%)	718 (14.5%)	93 (11.7%)	47 (15.7%)	5886 (15.9%)
1975-1979	4475 (15.4%)	305 (15.8%)	906 (18.3%)	156 (19.6%)	79 (26.4%)	5921 (16.0%)
1980-1984	8559 (29.5%)	717 (37.1%)	1576 (31.8%)	269 (33.8%)	87 (29.1%)	11 208 (30.3%)
1985-1989	5602 (19.3%)	517 (26.8%)	1149 (23.2%)	180 (22.6%)	26 (8.7%)	7474 (20.2%)
1990-1994	642 (2.2%)	61 (3.2%)	104 (2.1%)	33 (4.2%)	2 (0.7%)	842 (2.3%)
1995-1999	4 (0.0%)	2 (0.1%)	1 (0.0%)	0 (0.0%)	1 (0.3%)	8 (0.0%)
Race/ethnicity, *n* (%)						
Asian, non-Hispanic	465 (1.6%)	16 (0.8%)	50 (1.0%)	6 (0.8%)	5 (1.7%)	542 (1.5%)
Black, non-Hispanic	436 (1.5%)	37 (1.9%)	66 (1.3%)	12 (1.5%)	10 (3.3%)	561 (1.5%)
Hispanic	904 (3.1%)	91 (4.7%)	181 (3.6%)	38 (4.8%)	11 (3.7%)	1225 (3.3%)
Native American, non-Hispanic	75 (0.3%)	1 (0.1%)	10 (0.2%)	6 (0.8%)	0 (0%)	92 (0.2%)
White, non-Hispanic	26 297 (90.7%)	1709 (88.5%)	4410 (88.9%)	673 (84.7%)	255 (85.3%)	33 344 (90.2%)
Multiple/another, non-Hispanic	639 (2.2%)	65 (3.4%)	212 (4.3%)	55 (6.9%)	15 (5.0%)	986 (2.7%)
Missing	165 (0.6%)	12 (0.6%)	32 (0.6%)	5 (0.6%)	3 (1.0%)	217 (0.6%)
Region of residence at birth, *n* (%)						
Midwest	8408 (29.0%)	508 (26.3%)	1159 (23.4%)	160 (20.1%)	58 (19.4%)	10 293 (27.8%)
Northeast	6096 (21.0%)	459 (23.8%)	1079 (21.7%)	184 (23.1%)	71 (23.7%)	7889 (21.3%)
South	4011 (13.8%)	279 (14.4%)	656 (13.2%)	91 (11.4%)	55 (18.4%)	5092 (13.8%)
West	3833 (13.2%)	366 (19.0%)	882 (17.8%)	105 (13.2%)	23 (7.7%)	5209 (14.1%)
Outside of United States	1520 (5.2%)	107 (5.5%)	312 (6.3%)	40 (5.0%)	25 (8.4%)	2004 (5.4%)
Missing	5113 (17.6%)	212 (11.0%)	873 (17.6%)	215 (27.0%)	67 (22.4%)	6480 (17.5%)
Pregnancy intention, *n* (%)						
Actively trying/wanted then or sooner	19343 (66.7%)	1247 (64.6%)	3008 (60.6%)	430 (54.1%)	182 (60.9%)	24 210 (65.5%)
Not trying but glad	5496 (19.0%)	360 (18.6%)	798 (16.1%)	142 (17.9%)	36 (12.0%)	6832 (18.5%)
Wanted later but not then	3160 (10.9%)	226 (11.7%)	823 (16.6%)	138 (17.4%)	56 (18.7%)	4403 (11.9%)
Unwanted then and in future	982 (3.4%)	98 (5.1%)	332 (6.7%)	85 (10.7%)	25 (8.4%)	1522 (4.1%)
Induced abortions, *n* (%)						
No	27 304 (94.2%)	1759 (91.1%)	4344 (87.6%)	681 (85.7%)	258 (86.3%)	34 346 (92.9%)
Yes	1634 (5.6%)	166 (8.6%)	613 (12.4%)	112 (14.1%)	41 (13.7%)	2566 (6.9%)
Missing	43 (0.1%)	6 (0.3%)	4 (0.1%)	2 (0.3%)	0 (0.0%)	55 (0.1%)

Abbreviations: *n*, number; %, percentage.

a Descriptive characteristics in this table are reported at the level of individual pregnancies.

b In NHS3, the heterosexual with same-sex experience group consists of those who identified as completely heterosexual and either reported a prior SM identity or also reported having partners who were same-sex or nonbinary or being attracted to people of the same sex or nonbinary gender. In GUTS, the heterosexual with same-sex experience group consists of those who identified as completely heterosexual and also reported same-sex partners.

**Table 2:** Model-predicted probabilities and relative risk ratios of intention status of pregnancies by sexual orientation, Nurses’ Health Study 3 (NHS3) and Growing Up Today Study (GUTS).^a^

	Completely heterosexual with no same-sex experience		Completely heterosexual with same-sex experience			Mostly heterosexual			Bisexual			Lesbian/gay		
	*N*	Prob (%)	*N*	Prob (%)	RRR (95% CI)	*N*	Prob (%)	RRR (95% CI)	*N*	Prob (%)	RRR (95% CI)	*N*	Prob (%)	RRR (95% CI)
Actively trying/wanted then or sooner	19 343	68	1247	64	1.00	3008	61	1.00	430	52	1.00	182	69	1.00
Not trying but glad	5496	17	360	18	1.10 (0.87, 1.40)	798	15	0.97 (0.83, 1.13)	142	16	1.20 (0.83, 1.72)	36	7	0.40 (0.19, 0.81)
Wanted later but not then	3160	11	226	12	1.22 (0.95, 1.56)	823	16	1.67 (1.44, 1.95)	138	19	2.28 (1.65, 3.15)	56	14	1.23 (0.71, 2.13)
Unwanted then and in future	982	4	98	6	1.74 (1.18, 2.55)	332	7	2.40 (2.92, 3.01)	85	14	5.23 (3.52, 7.78)	25	10	2.94 (1.61, 5.36)

Abbreviations: *N*, number of pregnancies; Prob, predicted probability from multinomial models; RRR, relative risk ratio.

a Predicted probabilities and RRRs were estimated using multinomial models fit via generalized estimating equations with weights that were a product of stabilized inverse probability weights accounting for potential confounding by social origin (race/ethnicity, region of residence at birth, and year of birth of pregnant person) and inverse cluster size weights accounting for potential informative clustering.

**Table 3:** Model-predicted probabilities and risk ratios of induced abortions by sexual orientation stratified by pregnancy intention, Nurses’ Health Study 3 (NHS3) and Growing Up Today Study (GUTS).^a^, ^b^

	Completely heterosexual with no same-sex experience		Completely heterosexual with same-sex experience			Mostly heterosexual			Bisexual			Lesbian/gay		
	*N* abortions/*N* total pregnancies	Prob (%)	*N* abortions/*N* total pregnancies	Prob (%)	RR (95% CI)	*N* abortions/*N* total pregnancies	Prob (%)	RR (95% CI)	*N* abortions/*N* total pregnancies	Prob (%)	RR (95% CI)	*N* abortions/*N* total pregnancies	Prob (%)	RR (95% CI)
Actively trying/wanted then or sooner	74/19 317	0	9/1243	1	1.58 (0.73, 3.42)	23/3007	1	1.71 (1.02, 2.88)	5/430	1	2.09 (0.70, 6.21)	0/182	0	
Not trying but glad	33/5486	1	9/358	2	3.59 (1.55, 8.30)	14/797	2	3.11 (1.46, 6.60)	2/140	1		1/36	6	
Wanted later but not then	1084/3154	36	100/226	45	1.26 (1.03, 1.52)	380/821	44	1.25 (1.11, 1.40)	58/138	44	1.22 (0.94, 1.59)	27/56	49	1.38 (0.97, 1.96)
Unwanted then and in future	443/981	51	48/98	46	0.90 (0.67, 1.21)	196/332	62	1.22 (1.08, 1.39)	47/85	65	1.28 (1.03, 1.58)	13/25	49	0.97 (0.56, 1.70)

Abbreviations: *N*, number of pregnancies; Prob, predicted probability from log-linear models; RR, risk ratio.

a Predicted probabilities and RRs were estimated using log-linear models fit via generalized estimating equations with weights that were a product of stabilized inverse probability weights accounting for potential confounding by social origin (race/ethnicity, region of residence at birth, and year of birth of pregnant person) and inverse cluster size weights accounting for potential informative clustering.

b RRs not shown for cell sizes that have <5 observations.

## Data Availability

The data that support the findings of this study are available on request from the Channing Division of Network Medicine at Brigham and Women’s Hospital and Harvard Medical School (https://nurseshealthstudy.org/researchers). The data are not publicly available due to privacy and ethical restrictions.
