# Supplementary material for: Pregnancy intentions by sexual orientation among pregnancies across the life course
Source: Am J Epidemiol. Author manuscript; Available in PMC 2026 Aug 21. (PMC13496213; doi:10.1093/aje/kwag165)
Supplement: Supplementary file 1 [file NIHMS2204046-supplement-Supplement.docx]

**Supplementary Materials**

**Pregnancy intentions by sexual orientation among pregnancies across the life course**

Payal Chakraborty, Corinne H. Rocca, Colleen A. Reynolds, Isa Berzansky, Kodiak R.S. Soled, Sarah McKetta, Ange-Marie Hancock, Danielle Bessett, Sebastien Haneuse, Brittany M. Charlton

Table of Contents

[Figure S1. Pregnancy intention measurement in NHS3	2](#_Toc234313491)

[Table S1. Pregnancy intention questions in GUTS and NHS3	3](#_Toc234313492)

[Table S2. Missing data on pregnancy intention and type of missingness by sexual orientation for pregnancies in GUTS and NHS3	4](#_Toc234313493)

[Table S3. Characteristics of participants experiencing each pregnancy reported in the Nurses’ Health Study 3 (NHS3) and Growing Up Today Study (GUTS)	5](#_Toc234313494)

[Table S4. Crude relative risk ratios of intention status of pregnancies by sexual orientation, Nurses’ Health Study 3 (NHS3) and Growing Up Today Study (GUTS)	7](#_Toc234313495)

[Table S5. Distribution of pregnancy intentions by the number of years between pregnancy and reporting in the total sample and by sexual orientation	8](#_Toc234313496)

[Table S6. Relative risk ratios of intention status of pregnancies by sexual orientation adjusted for years between pregnancy occurance and reporting, Nurses’ Health Study 3 (NHS3) and Growing Up Today Study (GUTS)	9](#_Toc234313497)

[Table S7. Sexual orientation by birth year of pregnant person in Nurses’ Health Study 3 (NHS3) and Growing Up Today Study (GUTS)	10](#_Toc234313498)

# Figure S1. Pregnancy intention measurement in NHS3

# Table S1. Pregnancy intention questions in GUTS and NHS3

| Questionnaire | Question | Response Options |
| --- | --- | --- |
| NHS3 Questionnaire 1 Version 4 | Regarding this pregnancy, how did you feel about becoming pregnant? | 1. I was actively trying to become pregnant. 2. I was not actively trying but I was glad to become pregnant. 3. I wanted to become pregnant someday but not then. 4. I did not want to be pregnant then or at any time in the future. |
| NHS3 Questionnaire 1 Version 5 | Thinking back to when you got pregnant, which of the following best describes the timing of your pregnancy? | 1. I wanted to become pregnant at that time or sooner. 2. I was not actively trying but I was glad to become pregnant. 3. I wanted to be pregnant in the next 1-2 years but not yet. 4. I wanted to be pregnant in the next 2+ years but not yet. 5. I did not want to be pregnant then or at any time. |
| NHS3 Questionnaire 13 | Thinking back to when you got pregnant, which of the following best describes the timing of your pregnancy? | 1. I wanted to become pregnant at that time or sooner. 2. I was not actively trying but I was glad to become pregnant. 3. I wanted to be pregnant in the next 1-2 years but not yet. 4. I wanted to be pregnant in the next 2+ years but not yet. 5. I did not want to be pregnant then or at any time. |
| NHS3 Catch-Up Questionnaire | Regarding this pregnancy, how did you feel about becoming pregnant? | 1. I was actively trying to become pregnant. 2. I was not actively trying but I was glad to become pregnant. 3. I wanted to become pregnant someday but not then. 4. I did not want to be pregnant then or at any time in the future. |
| GUTS | Regarding this pregnancy, my partner and I: | 1. Were actively trying to become pregnant. 2. Were not actively trying, but I was glad to become pregnant. 3. Wanted to become pregnant someday, but not then. 4. Did not want to be pregnant then or at any time in the future. |

# Table S2. Missing data on pregnancy intention and type of missingness by sexual orientation for pregnancies in GUTS and NHS3

|  | Completely heterosexual with no same-sex experience | Completely heterosexual with same-sex experience | Mostly heterosexual | Bisexual | Lesbian/gay |  |  |
| --- | --- | --- | --- | --- | --- | --- | --- |
|  | GUTS | | | | | |  |
| Pregnancy intention, n (%) |  |  |  |  |  |  |  |
| Actively trying/wanted then or sooner | 4,084 (69.9%) | 253 (64.4%) | 676 (61.3%) | 57 (37.7%) | 25 (78.1%) |  |  |
| Not trying but glad | 1,047 (17.9%) | 79 (20.1%) | 177 (16.0%) | 30 (19.9%) | 0 (0.0%) |  |  |
| Wanted later but not then | 566 (9.7%) | 47 (12.0%) | 169 (15.3%) | 39 (25.8%) | 4 (12.5%) |  |  |
| Unwanted then and in future | 145 (2.5%) | 14 (3.6%) | 80 (7.3%) | 24 (15.9%) | 3 (9.4%) |  |  |
| Missing due to non-response | 4 (0.1%) | 0 (0%) | 1 (0.1%) | 1 (0.7%) | 0 (0.0%) |  |  |
|  | NHS3 | | | | | |  |
| Pregnancy intention, n (%) |  |  |  |  |  |  |  |
| Actively trying/wanted then or sooner | 15,259 (65.7%) | 994 (64.5%) | 2,332 (60.3%) | 373 (57.7%) | 157 (58.6%) |  |  |
| Not trying but glad | 4,449 (19.2%) | 281 (18.2%) | 621 (16.1%) | 112 (17.3%) | 36 (13.4%) |  |  |
| Wanted later but not then | 2,594 (11.2%) | 179 (11.6%) | 654 (16.9%) | 99 (15.3%) | 52 (19.4%) |  |  |
| Unwanted then and in future | 837 (3.6%) | 84 (5.5%) | 252 (6.5%) | 61 (9.4%) | 22 (8.2%) |  |  |
| Missing due to non-response | 83 (0.4%) | 2 (0.1%) | 7 (0.2%) | 1 (0.2%) | 1 (0.4%) |  |  |
| Missing due to not being assessed^b^ | 9,674 | 351 | 1,270 | 273 | 97 |  |  |
| n=number; %=percentage; SD=standard deviation  ^a^ These represent missing values from the prospective collection of pregnancies, which only had a binary measure of intention.  ^b^ In NHS3, pregnancy intention was measured starting for pregnancies in the fourth version of the first questionnaire as well as in the thirteenth questionnaire. Later respondents who answered the first three versions of the first questionnaire were asked to complete a catch-up questionnaire where they were asked about intentions only if they confirmed details about their previous pregnancies. These are missing because respondents never completed the catch-up questionnaire, or they completed the catch-up questionnaire and were not given the intention question. See Supplemental Figure 1 for more details. | | | | | | | |

Table S3. Characteristics of participants experiencing each pregnancy reported in the Nurses’ Health Study 3 (NHS3) and Growing Up Today Study (GUTS)^a^

|  | Completely heterosexual with no same-sex experience | Completely heterosexual with same-sex experience^b^ | Mostly heterosexual | Bisexual | Lesbian/gay | Overall |
| --- | --- | --- | --- | --- | --- | --- |
|  | GUTS | | | | | |
|  | (n=5,842, %=77.7) | (n=393, %=5.2) | (n=1,102, %=14.7) | (n=150, %=2.0) | (n=32, %=0.4) | (n=7,519, %=100.0) |
| Birth year of pregnant person, n(%) |  |  |  |  |  |  |
| 1980-1984 | 3,157 (54.0%) | 244 (62.1%) | 583 (52.9%) | 66 (44.0%) | 19 (59.4%) | 4,069 (54.1%) |
| 1985-1989 | 2,374 (40.6%) | 142 (36.1%) | 471 (42.7%) | 69 (46.0%) | 13 (40.6%) | 3,069 (40.8%) |
| 1990 | 311 (5.3%) | 7 (1.8%) | 48 (4.4%) | 15 (10.0%) | 0 (0%) | 381 (5.1%) |
| Race/ethnicity, n (%) |  |  |  |  |  |  |
| Asian, non-Hispanic | 36 (0.6%) | 0 (0%) | 8 (0.7%) | 0 (0%) | 0 (0%) | 44 (0.6%) |
| Black, non-Hispanic | 16 (0.3%) | 5 (1.3%) | 13 (1.2%) | 0 (0%) | 0 (0%) | 34 (0.5%) |
| Hispanic | 86 (1.5%) | 29 (7.4%) | 8 (0.7%) | 8 (5.3%) | 1 (3.1%) | 132 (1.8%) |
| Native American, non-Hispanic | 10 (0.2%) | 0 (0%) | 1 (0.1%) | 1 (0.7%) | 0 (0%) | 12 (0.2%) |
| White, non-Hispanic | 5,499 (94.1%) | 337 (85.8%) | 1,019 (92.5%) | 133 (88.7%) | 27 (84.4%) | 7,015 (93.3%) |
| Multiple/another, non-Hispanic | 159 (2.7%) | 13 (3.3%) | 47 (4.3%) | 8 (5.3%) | 4 (12.5%) | 231 (3.1%) |
| Missing | 36 (0.6%) | 9 (2.3%) | 6 (0.5%) | 0 (0%) | 0 (0%) | 51 (0.7%) |
| Region of residence at birth, n (%) |  |  |  |  |  |  |
| Midwest | 2,400 (41.1%) | 131 (33.3%) | 400 (36.3%) | 65 (43.3%) | 9 (28.1%) | 3,005 (40.0%) |
| Northeast | 1,821 (31.2%) | 127 (32.3%) | 370 (33.6%) | 41 (27.3%) | 10 (31.3%) | 2,369 (31.5%) |
| South | 826 (14.1%) | 58 (14.8%) | 131 (11.9%) | 19 (12.7%) | 7 (21.9%) | 1,041 (13.8%) |
| West | 792 (13.6%) | 77 (19.6%) | 201 (18.2%) | 25 (16.7%) | 6 (18.8%) | 1,101 (14.6%) |
| Outside of US | 0 (0.0%) | 0 (0.0%) | 0 (0.0%) | 0 (0.0%) | 0 (0.0%) | 0 (0.0%) |
| Missing | 3 (0.1%) | 0 (0%) | 0 (0%) | 0 (0%) | 0 (0%) | 3 (0.0%) |
| Pregnancy intention, n (%) |  |  |  |  |  |  |
| Actively trying/wanted then or sooner | 4,084 (69.9%) | 253 (64.4%) | 676 (61.3%) | 57 (38.0%) | 25 (78.1%) | 5,095 (67.8%) |
| Not trying but glad | 1047 (17.9%) | 79 (20.1%) | 177 (16.1%) | 30 (20.0%) | 0 (0%) | 1,333 (17.7%) |
| Wanted later but not then | 566 (9.7%) | 47 (12.0%) | 169 (15.3%) | 39 (26.0%) | 4 (12.5%) | 825 (11.0%) |
| Unwanted then and in future | 145 (2.5%) | 14 (3.6%) | 80 (7.3%) | 24 (16.0%) | 3 (9.4%) | 266 (3.5%) |
| Induced abortions, n(%) |  |  |  |  |  |  |
| No | 5,516 (94.4%) | 355 (90.3%) | 973 (88.3%) | 120 (80.0%) | 31 (96.9%) | 6,995 (93.0%) |
| Yes | 310 (5.3%) | 35 (8.9%) | 128 (11.6%) | 29 (19.3%) | 1 (3.1%) | 503 (6.7%) |
| Missing | 16 (0.3%) | 3 (0.8%) | 1 (0.1%) | 1 (0.7%) | 0 (0%) | 21 (0.3%) |
|  | NHS3 | | | | | |
|  | (n=23,139, %=78.6) | (n=1,538, %=5.2) | (n=3,859, %=13.1) | (n=645, %=2.2) | (n=267, %=0.9) | (n=29,548, %=100.0) |
| Birth year of pregnant person, n(%) |  |  |  |  |  |  |
| 1965-1969 | 4,857 (21.0%) | 143 (9.3%) | 507 (13.1%) | 64 (9.9%) | 57 (21.3%) | 5,628 (19.1%) |
| 1970-1974 | 4,842 (20.9%) | 186 (12.1%) | 718 (18.6%) | 93 (14.4%) | 47 (17.6%) | 5,886 (20.0%) |
| 1975-1979 | 4,475 (19.3%) | 305 (19.8%) | 906 (23.5%) | 156 (24.2%) | 79 (29.6%) | 5,921 (20.1%) |
| 1980-1984 | 5,402 (23.3%) | 473 (30.8%) | 993 (25.7%) | 203 (31.5%) | 68 (25.5%) | 7,139 (24.2%) |
| 1985-1989 | 3,228 (14.0%) | 375 (24.4%) | 678 (17.6%) | 111 (17.2%) | 13 (4.9%) | 4,405 (15.0%) |
| 1990-1994 | 331 (1.4%) | 54 (3.5%) | 56 (1.5%) | 18 (2.8%) | 2 (0.7%) | 461 (1.6%) |
| 1995-1999 | 4 (0.0%) | 2 (0.1%) | 1 (0.0%) | 0 (0%) | 1 (0.4%) | 8 (0.0%) |
| Race/ethnicity, n (%) |  |  |  |  |  |  |
| Asian, non-Hispanic | 429 (1.9%) | 16 (1.0%) | 42 (1.1%) | 6 (0.9%) | 5 (1.9%) | 498 (1.7%) |
| Black, non-Hispanic | 420 (1.8%) | 32 (2.1%) | 53 (1.4%) | 12 (1.9%) | 10 (3.7%) | 527 (1.8%) |
| Hispanic | 818 (3.5%) | 62 (4.0%) | 173 (4.5%) | 30 (4.7%) | 10 (3.7%) | 1,093 (3.7%) |
| Native American, non-Hispanic | 65 (0.3%) | 1 (0.1%) | 9 (0.2%) | 5 (0.8%) | 0 (0%) | 80 (0.3%) |
| White, non-Hispanic | 20,798 (89.9%) | 1,372 (89.2%) | 3,391 (87.9%) | 540 (83.7%) | 228 (85.4%) | 26,329 (89.4%) |
| Multiple/another, non-Hispanic | 480 (2.1%) | 52 (3.4%) | 165 (4.3%) | 47 (7.3%) | 11 (4.1%) | 755 (2.6%) |
| Missing | 129 (0.6%) | 3 (0.2%) | 26 (0.7%) | 5 (0.8%) | 3 (1.1%) | 166 (0.6%) |
| Region of residence at birth, n (%) |  |  |  |  |  |  |
| Midwest | 6,008 (26.0%) | 377 (24.5%) | 759 (19.7%) | 95 (14.7%) | 49 (18.4%) | 7,288 (24.7%) |
| Northeast | 4,275 (18.5%) | 332 (21.6%) | 709 (18.4%) | 143 (22.2%) | 61 (22.8%) | 5,520 (18.7%) |
| South | 3,185 (13.8%) | 221 (14.4%) | 525 (13.6%) | 72 (11.2%) | 48 (18.0%) | 4,051 (13.8%) |
| West | 3,041 (13.1%) | 289 (18.8%) | 681 (17.6%) | 80 (12.4%) | 17 (6.4%) | 4,108 (14.0%) |
| Outside of US | 1,520 (6.6%) | 107 (7.0%) | 312 (8.1%) | 40 (6.2%) | 25 (9.4%) | 2,004 (6.8%) |
| Missing | 5,110 (22.1%) | 212 (13.8%) | 873 (22.6%) | 215 (33.3%) | 67 (25.1%) | 6,477 (22.0%) |
| Pregnancy intention, n (%) |  |  |  |  |  |  |
| Actively trying/wanted then or sooner | 15,259 (65.9%) | 994 (64.6%) | 2,332 (60.4%) | 373 (57.8%) | 157 (58.8%) | 19,115 (64.9%) |
| Not trying but glad | 4,449 (19.2%) | 281 (18.3%) | 621 (16.1%) | 112 (17.4%) | 36 (13.5%) | 5,499 (18.7%) |
| Wanted later but not then | 2,594 (11.2%) | 179 (11.6%) | 654 (16.9%) | 99 (15.3%) | 52 (19.5%) | 3,578 (12.2%) |
| Unwanted then and in future | 837 (3.6%) | 84 (5.5%) | 252 (6.5%) | 61 (9.5%) | 22 (8.2%) | 1,256 (4.3%) |
| Induced abortions, n(%) |  |  |  |  |  |  |
| No | 21,788 (94.2%) | 1,404 (91.3%) | 3,371 (87.4%) | 561 (87.0%) | 227 (85.0%) | 27,351 (92.9%) |
| Yes | 1,324 (5.7%) | 131 (8.5%) | 485 (12.6%) | 83 (12.9%) | 40 (15.0%) | 2,063 (7.0%) |
| Missing | 27 (0.1%) | 3 (0.2%) | 3 (0.1%) | 1 (0.2%) | 0 (0%) | 34 (0.1%) |
| n=number; %=percentage; SD=standard deviation  ^a^ Descriptive characteristics in this table are reported at the level of individual pregnancies.  ^b^ In NHS3, the heterosexual with same-sex experience group consists of those who identified as completely heterosexual and either reported a prior SM identity or also reported having partners who were same-sex or non-binary or being attracted to people of the same sex or non-binary gender. In GUTS, the heterosexual with same-sex experience group consists of those who identified as completely heterosexual and also reported same-sex partners. | | | | | | |

Table S4. Crude relative risk ratios of intention status of pregnancies by sexual orientation, Nurses’ Health Study 3 (NHS3) and Growing Up Today Study (GUTS)^a^

|  | Completely heterosexual with no same-sex experience | |  | Completely heterosexual with same-sex experience | | |  | Mostly heterosexual | | |  | Bisexual | | |  | Lesbian/gay | | |
| --- | --- | --- | --- | --- | --- | --- | --- | --- | --- | --- | --- | --- | --- | --- | --- | --- | --- | --- |
|  | N | Prob |  | N | Risk | RRR (95% CI) |  | N | Prob | RRR (95% CI) |  | N | Prob | RRR (95% CI) |  | N | Prob | RRR (95% CI) |
| Actively trying/wanted then or sooner | 19,343 | 68% |  | 1,247 | 67% | 1.00 |  | 3,008 | 62% | 1.00 |  | 430 | 52% | 1.00 |  | 182 | 66% | 1.00 |
| Not trying but glad | 5,496 | 17% |  | 360 | 16% | 0.96 (0.76, 1.21) |  | 798 | 14% | 0.90 (0.77, 1.06) |  | 142 | 15% | 1.09 (0.76, 1.56) |  | 36 | 8% | 0.50 (0.26, 0.97) |
| Wanted later but not then | 3,160 | 11% |  | 226 | 12% | 1.08 (0.84, 1.39) |  | 823 | 16% | 1.59 (1.36, 1.85) |  | 138 | 20% | 2.38 (1.74, 3.27) |  | 56 | 16% | 1.46 (0.86, 2.47) |
| Unwanted then and in future | 982 | 4% |  | 98 | 5% | 1.47 (1.00, 2.16) |  | 332 | 7% | 2.30 (1.84, 2.88) |  | 85 | 13% | 4.97 (3.36, 7.34) |  | 25 | 10% | 2.88 (1.52, 5.46) |
| N=number of pregnancies; RRR= relative risk ratio; CI=confidence interval; Prob=predicted probability from multinomial models  ^a^ Risks and RRRs were estimated using multinomial models fit via generalized estimating equations with inverse cluster size weights accounting for potential informative clustering. | | | | | | | | | | | | | | | | | | |

# Table S5. Distribution of pregnancy intentions by the number of years between pregnancy and reporting in the total sample and by sexual orientation

|  | Years between pregancy and reporting | Pregnancy intention | | | |
| --- | --- | --- | --- | --- | --- |
|  |  | Actively trying/wanted then or sooner | Not trying but glad | Wanted later but not then | Unwanted then and in future |
| Total sample | 0 years | 607 (76.5%) | 127 (16%) | 39 (4.9%) | 20 (2.5%) |
|  | 1-5 years | 8442 (77.7%) | 1634 (15%) | 498 (4.6%) | 284 (2.6%) |
|  | 6-10 years | 7666 (72.1%) | 1855 (17.4%) | 758 (7.1%) | 353 (3.3%) |
|  | 11-20 years | 5457 (58.2%) | 1967 (21%) | 1461 (15.6%) | 484 (5.2%) |
|  | >20 years | 1513 (34.4%) | 1034 (23.5%) | 1504 (34.2%) | 345 (7.8%) |
| Completely heterosexual with no same-sex experience | 0 years | 398 (76.2%) | 94 (18%) | 21 (4%) | 9 (1.7%) |
|  | 1-5 years | 6213 (78.2%) | 1227 (15.4%) | 341 (4.3%) | 168 (2.1%) |
|  | 6-10 years | 6298 (73.7%) | 1507 (17.6%) | 513 (6%) | 228 (2.7%) |
|  | 11-20 years | 4664 (61.4%) | 1608 (21.2%) | 1031 (13.6%) | 298 (3.9%) |
|  | >20 years | 1337 (36.8%) | 887 (24.4%) | 1162 (31.9%) | 252 (6.9%) |
| Completely heterosexual with same-sex experience | 0 years | 72 (74.2%) | 13 (13.4%) | 9 (9.3%) | 3 (3.1%) |
|  | 1-5 years | 698 (74.6%) | 160 (17.1%) | 45 (4.8%) | 33 (3.5%) |
|  | 6-10 years | 312 (68.6%) | 84 (18.5%) | 38 (8.4%) | 21 (4.6%) |
|  | 11-20 years | 110 (40.6%) | 61 (22.5%) | 76 (28%) | 24 (8.9%) |
|  | >20 years | 32 (25%) | 30 (23.4%) | 51 (39.8%) | 15 (11.7%) |
| Mostly heterosexual | 0 years | 104 (78.2%) | 17 (12.8%) | 7 (5.3%) | 5 (3.8%) |
|  | 1-5 years | 1291 (77.7%) | 215 (12.9%) | 90 (5.4%) | 65 (3.9%) |
|  | 6-10 years | 886 (65.2%) | 225 (16.6%) | 167 (12.3%) | 80 (5.9%) |
|  | 11-20 years | 547 (46.4%) | 225 (19.1%) | 282 (23.9%) | 125 (10.6%) |
|  | >20 years | 127 (24.7%) | 94 (18.3%) | 240 (46.7%) | 53 (10.3%) |
| Bisexual | 0 years | 25 (78.1%) | 3 (9.4%) | 2 (6.2%) | 2 (6.2%) |
|  | 1-5 years | 181 (72.7%) | 31 (12.4%) | 19 (7.6%) | 18 (7.2%) |
|  | 6-10 years | 115 (55%) | 37 (17.7%) | 37 (17.7%) | 20 (9.6%) |
|  | 11-20 years | 89 (40.6%) | 51 (23.3%) | 51 (23.3%) | 28 (12.8%) |
|  | >20 years | 9 (14.1%) | 15 (23.4%) | 25 (39.1%) | 15 (23.4%) |
| Lesbian/gay | 0 years | 8 (88.9%) | 0 (0%) | 0 (0%) | 1 (11.1%) |
|  | 1-5 years | 59 (93.7%) | 1 (1.6%) | 3 (4.8%) | 0 (0%) |
|  | 6-10 years | 55 (85.9%) | 2 (3.1%) | 3 (4.7%) | 4 (6.2%) |
|  | 11-20 years | 47 (47.5%) | 22 (22.2%) | 21 (21.2%) | 9 (9.1%) |
|  | >20 years | 8 (15.4%) | 8 (15.4%) | 26 (50%) | 10 (19.2%) |

Table S6. Relative risk ratios of intention status of pregnancies by sexual orientation adjusted for years between pregnancy occurance and reporting, Nurses’ Health Study 3 (NHS3) and Growing Up Today Study (GUTS)^a^

|  | Completely heterosexual with no same-sex experience |  | Completely heterosexual with same-sex experience | |  | Mostly heterosexual | |  | Bisexual | |  | Lesbian/gay | |
| --- | --- | --- | --- | --- | --- | --- | --- | --- | --- | --- | --- | --- | --- |
|  | N |  | N | RRR (95% CI) |  | N | RRR (95% CI) |  | N | RRR (95% CI) |  | N | RRR (95% CI) |
| Actively trying/wanted then or sooner | 19,343 |  | 1,247 | 1.00 |  | 3,008 | 1.00 |  | 430 | 1.00 |  | 182 | 1.00 |
| Not trying but glad | 5,496 |  | 360 | 1.22 (0.96, 1.55) |  | 798 | 1.03 (0.88, 1.20) |  | 142 | 1.26 (0.87, 1.81) |  | 36 | 0.39 (0.19, 0.80) |
| Wanted later but not then | 3,160 |  | 226 | 1.48 (1.14, 1.94) |  | 823 | 1.86 (1.58, 2.20) |  | 138 | 2.59 (1.81, 3.71) |  | 56 | 1.19 (0.69, 2.06) |
| Unwanted then and in future | 982 |  | 98 | 2.01 (1.35, 2.99) |  | 332 | 2.69 (2.13, 3.38) |  | 85 | 5.82 (3.87, 8.76) |  | 25 | 2.98 (1.63, 5.45) |
| N=number of pregnancies; RRR=relative risk ratio; CI=confidence interval; Prob=predicted probability from multinomial models  ^a^ Predicted probabilities and RRRs were estimated using multinomial models fit via generalized estimating equations with weights that were a product of stabilized inverse probability weights accounting for potential confounding by social origin (race/ethnicity, region of residence at birth, year of birth of pregnant person) and inverse cluster size weights accounting for potential informative clustering. Models also include adjustment for years between pregnancy occurance and reporting. | | | | | | | | | | | | | |

# Table S7. Sexual orientation by birth year of pregnant person in Nurses’ Health Study 3 (NHS3) and Growing Up Today Study (GUTS)

|  | Sexual orientation | | | | |
| --- | --- | --- | --- | --- | --- |
| Birth year of pregnant person | Completely heterosexual with no same-sex experience | Completely heterosexual with same-sex experience | Mostly heterosexual | Bisexual | Lesbian/gay |
| 1965-1969 | 4857 (86.3%) | 143 (2.5%) | 507 (9%) | 64 (1.1%) | 57 (1%) |
| 1970-1974 | 4842 (82.3%) | 186 (3.2%) | 718 (12.2%) | 93 (1.6%) | 47 (0.8%) |
| 1975-1979 | 4475 (75.6%) | 305 (5.2%) | 906 (15.3%) | 156 (2.6%) | 79 (1.3%) |
| 1980-1984 | 8559 (76.4%) | 717 (6.4%) | 1576 (14.1%) | 269 (2.4%) | 87 (0.8%) |
| 1985-1989 | 5602 (75%) | 517 (6.9%) | 1149 (15.4%) | 180 (2.4%) | 26 (0.3%) |
| 1990-1994 | 642 (76.2%) | 61 (7.2%) | 104 (12.4%) | 33 (3.9%) | 2 (0.2%) |
| 1995-1999 | 4 (50%) | 2 (25%) | 1 (12.5%) | 0 (0%) | 1 (12.5%) |
